# Increasing Amerindian diversity in AD genetics research, A Peruvian perspective

**DOI:** 10.1002/alz.094392

**Published:** 2025-01-09

**Authors:** Mario Cornejo‐Olivas, Maryenela Z. Illanes‐Manrique

**Affiliations:** ^1^ Neurogenetics Working Group, Universidad Científica del Sur, Lima Peru

## Abstract

Amerindian (AI) populations are substantially underrepresented in AD genetic studies. The Alzheimer’s Disease Sequencing Project (ADSP), a global genetic initiative established by the National Institute of Aging (NIA) is supporting regional initiatives in Latin America and its admixed population. Latin America is the largest recently admixed population, with variable Native American, European, and African ancestry proportions, as result of successive settlements and new massive migrations. The Native American population from Latin America are descendants of ancestral Asians who entered America through Beringia 15 000 years ago. By the 14‐15 th century the Native American population was about tens of millions with uneven distribution across the Americas. However, about 90% of the NA populated collapsed due to violence, famine, and infectious diseases. Consequently, the NA from Latin America exhibit lower genetic diversity with higher differences among countries. Peru is the fifth largest country of South America with a complex geography that comprise the Andes mountains, the Amazon jungle, and the Pacific coast. Peru is an emergent economy dealing with governance disparities, centralism in Lima, the capital city, with notable inequities in income distribution. The “modern mestizo “population in Peru concentrates a high proportion (˜70%) of NA ancestry, mostly derived from the ancient pre‐Inca and Inca civilizations. Over the past decade, both local and collaborative efforts, have been contributing to the AD genetic research in the Peruvian populations. A combination of regional research teams and multisource ascertainment approaches allowed the recruitment of 250 AD patients and 400 cognitively unimpaired controls from five different regions across Peru (source: PeADI initiative). The studies performed in the Peruvian cohort have been demonstrating the important role of the Amerindian ancestry in the genetic architecture of AD, including the estimation of APOE4 risk higher than non‐Hispanic Whites and Africans, and the recent identification a novel SORL1 variant in a Peruvian AD family. Despite these significant contributions, performing research in Latin American regions requires strong local brain health programs with community engaged approaches to prevent disparities and contributing to long‐term local capacity building.

